# Supplementary material for: Evaluation of Density-Based Spatial Clustering for Identifying Genomic Loci Associated with Ischemic Stroke in Genome-Wide Data
Source: Int J Mol Sci. 2023 Oct 19;24(20):15355. doi: 10.3390/ijms242015355 (PMC10607504; doi:10.3390/ijms242015355)
Supplement: Supplementary file 1 [file ijms-24-15355-s001.zip › supplementary figures.pdf]

Supplementary Materials

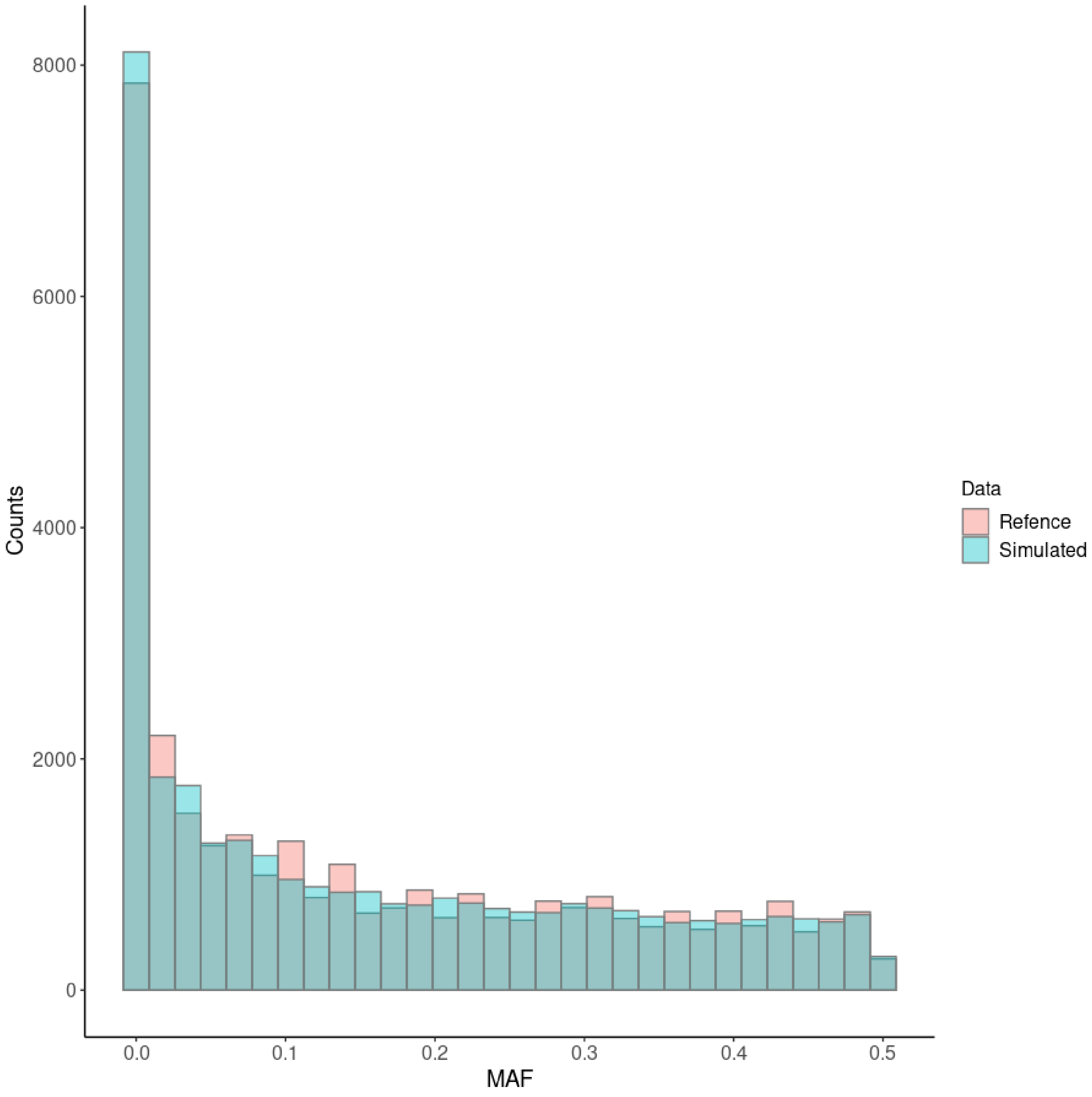

Figure S1. The histograms of minor allele frequencies at chromosome 22 for reference and simulated datasets. The number of SNPs is 3208. The sizes of populations are 99 (reference) and 5581 (simulated data).

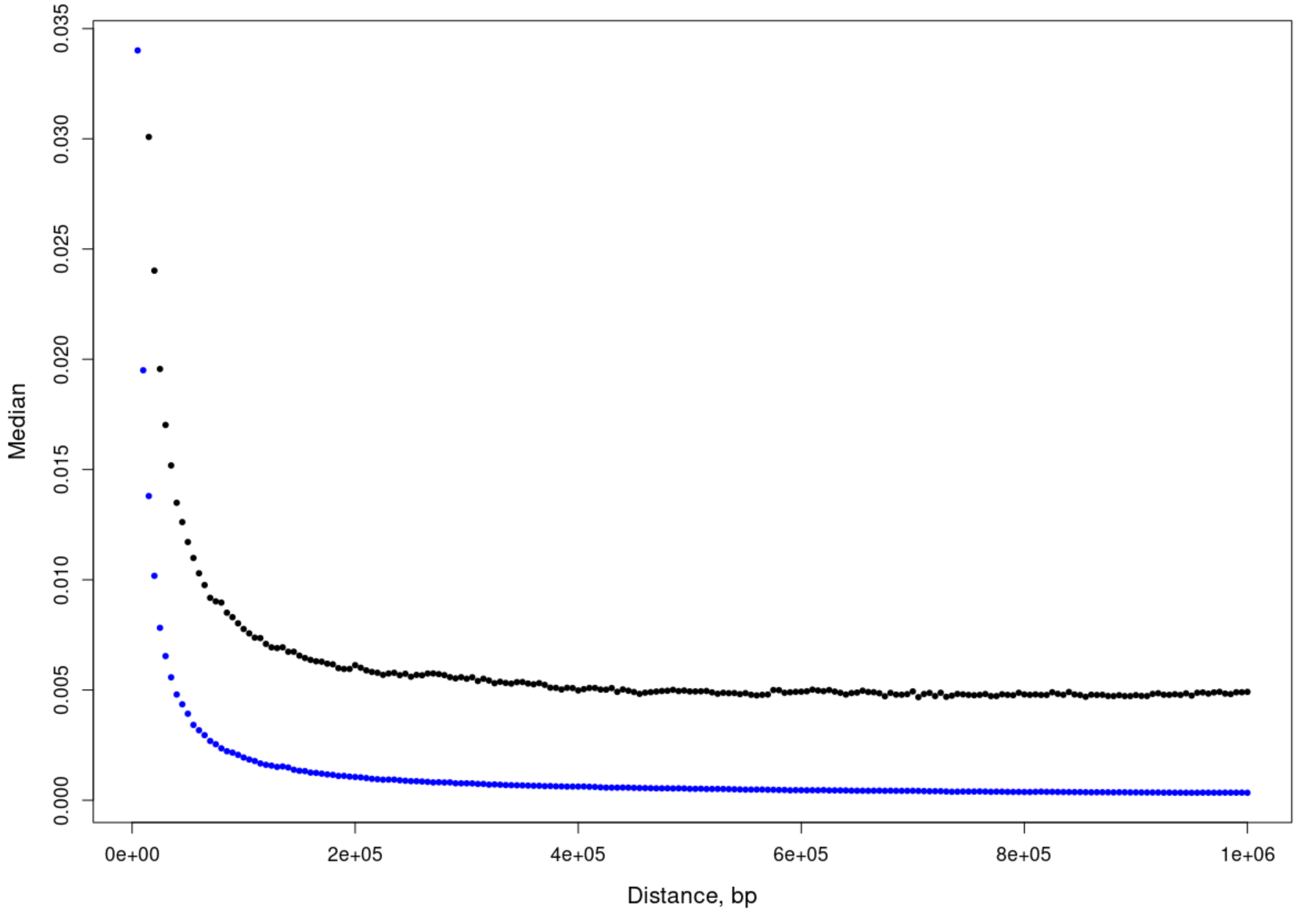

Figure S2. The decay of LD. The Y axis shows the median values of r^2 in the windows of 5000 bp on the distance up to 1M bp for reference panel (black) and simulated data (blue).

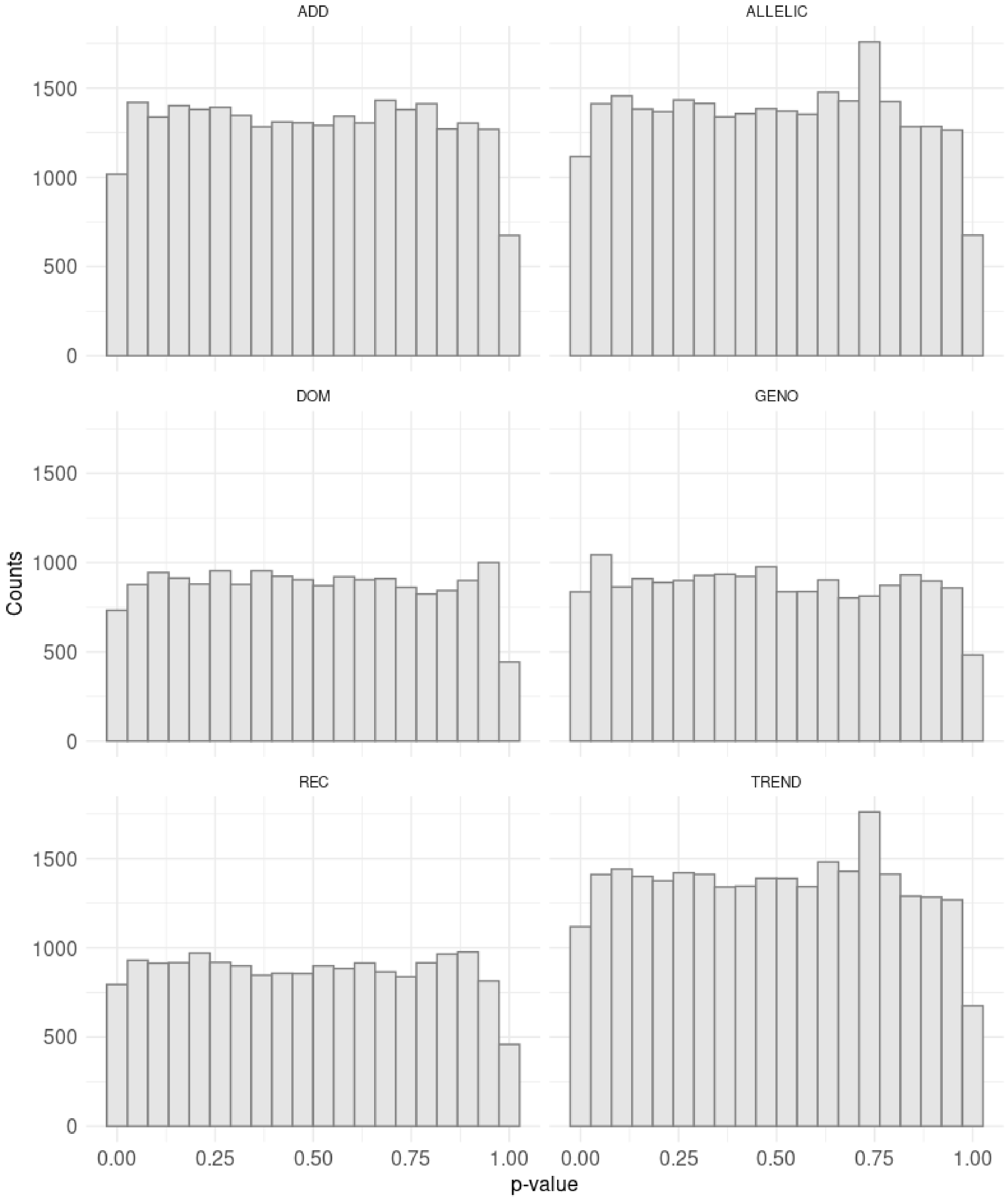

Figure S3. The distribution of p-values obtained for simulated data with chi-square test of independence (dominant (DOM), recessive (REC), and genotypic models (GENO) of inheritance), Fisher's exact test (ALLELIC), Cochran-Armitage trend test (TREND) and logistic regression (ADD). The tests were made with Plink 1.9.

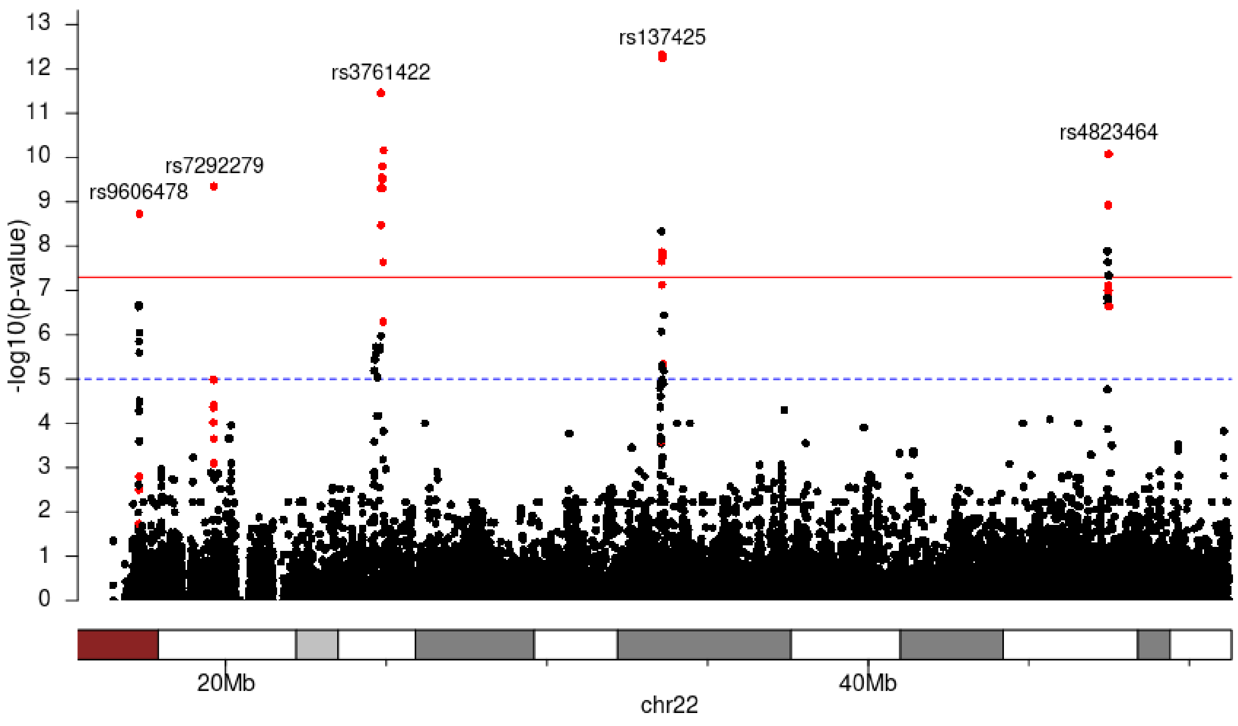

Figure S4. Manhattan plot for ALLELIC test made for synthetic dataset. The five disease SNPs are annotated by their ids. The blue line denotes the p-value of 1e-05 and the red one – 5e-08. The SNPs from blocks associated significantly with the phenotype according to haplotype tests are marked by red color.

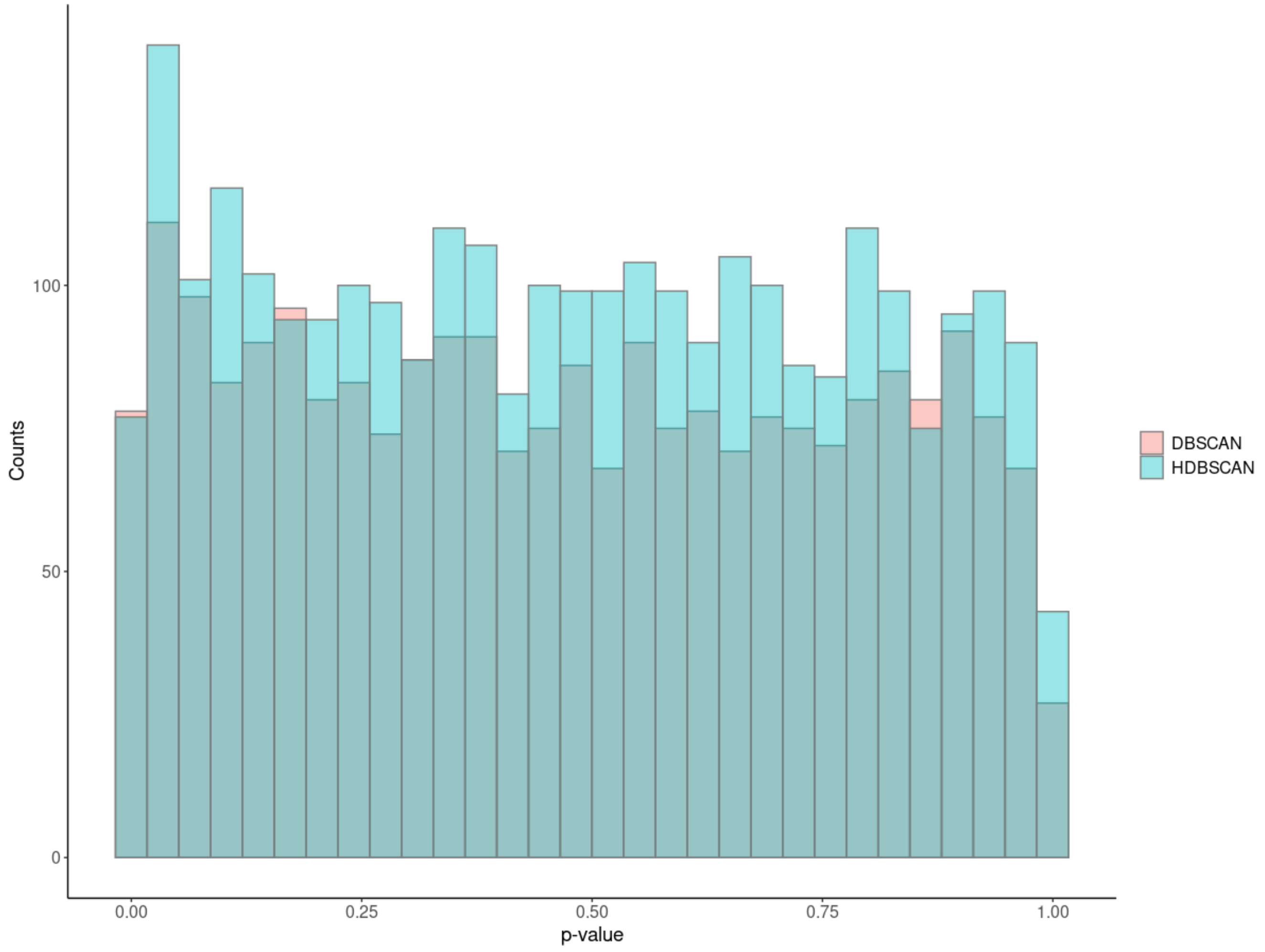

Figure S5. The distribution of p-values obtained by the haplotype tests applied to blocks corresponding to the clusters identified with DBSCAN and HDBSCAN algorithms in simulated dataset.

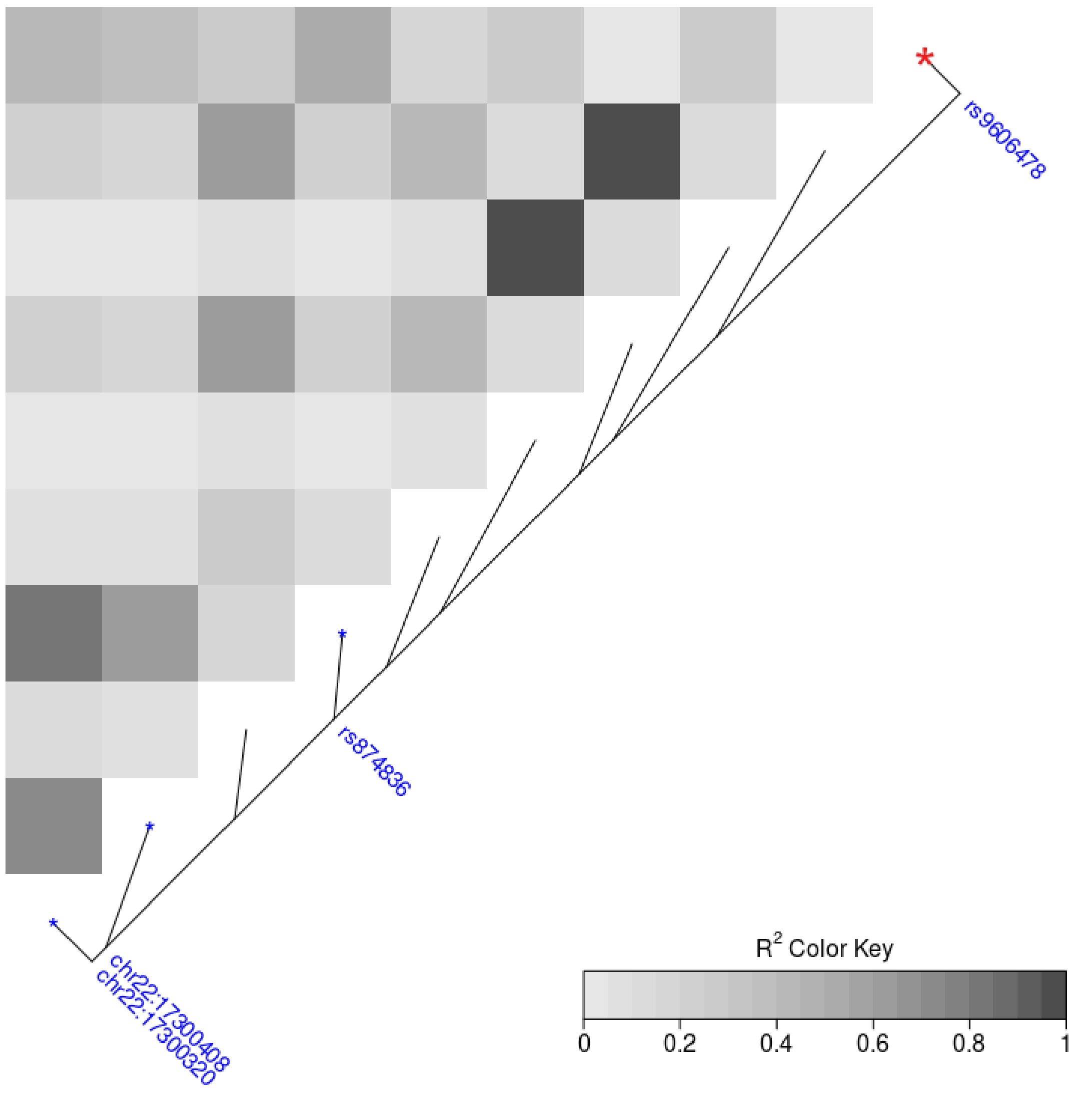

Figure S6. The LD heatmap of the genome region involving LD-block of 4 SNPs including the disease one (rs9606478). The SNPs of LD-block are marked by blue color, the disease SNP is marked by red asterisk.

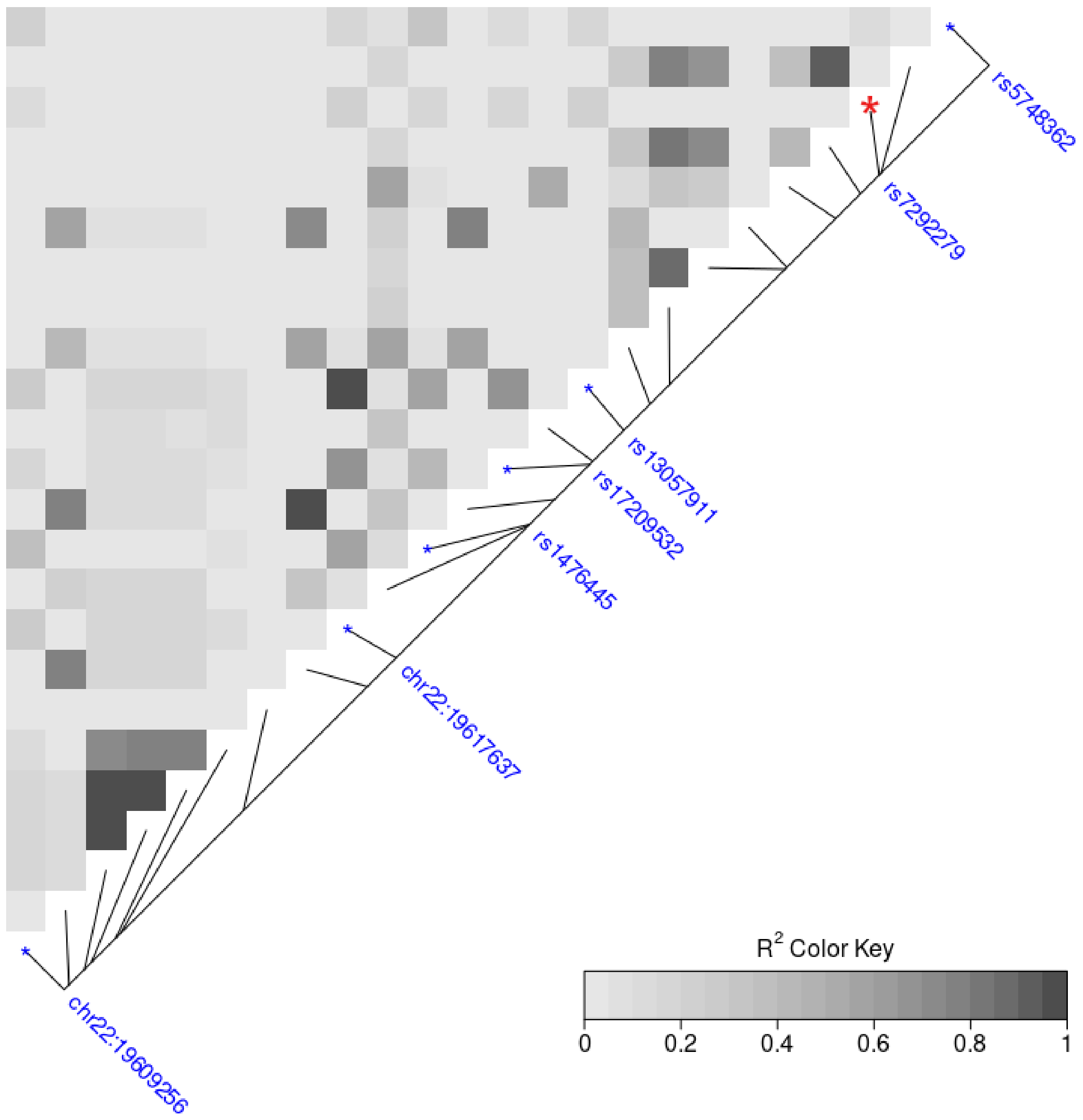

Figure S7. The LD heatmap of the genome region involving LD-block of 7 SNPs including the disease one (rs7292279). The SNPs of LD-block are marked by blue color, the disease SNP is marked by red asterisk.

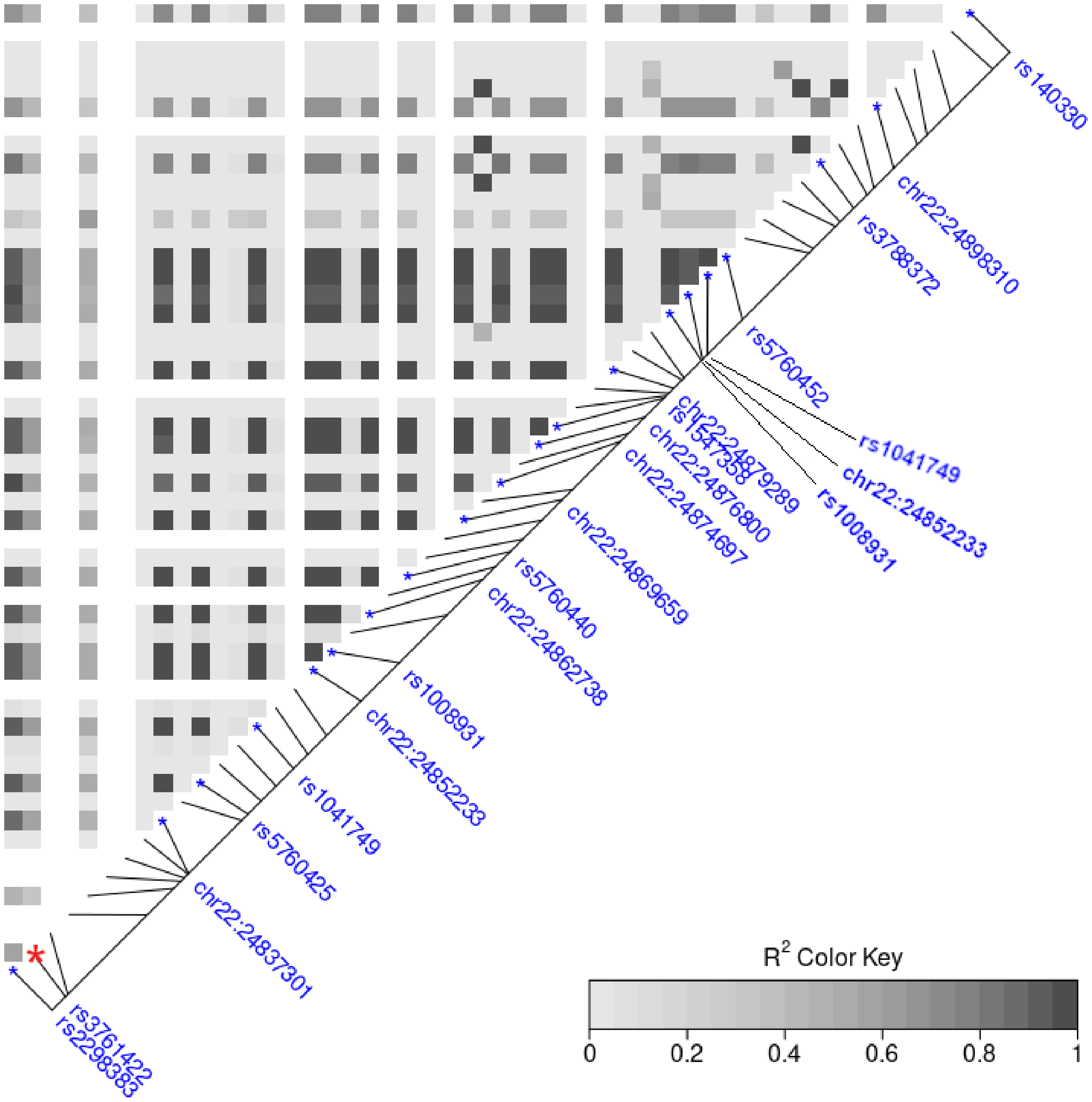

Figure S8. The LD heatmap of the genome region involving LD-block of 21 SNPs including the disease one (rs3761422). The SNPs of LD-block are marked by blue color, the disease SNP is marked by red asterisk.

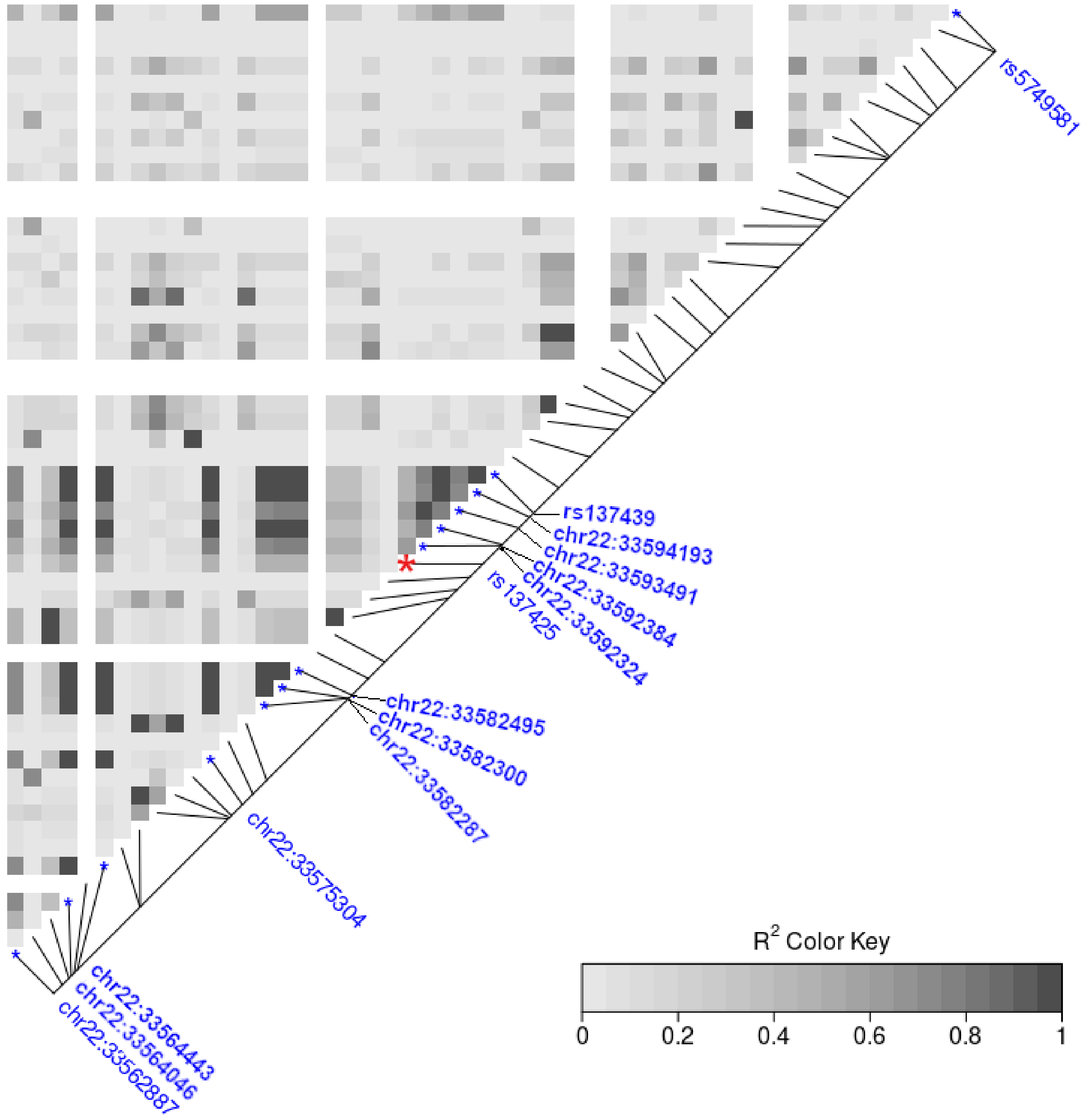

Figure S9. The LD heatmap of the genome region involving LD-block of 14 SNPs including the disease one (rs137425). The SNPs of LD-block are marked by blue color, the disease SNP is marked by red asterisk.

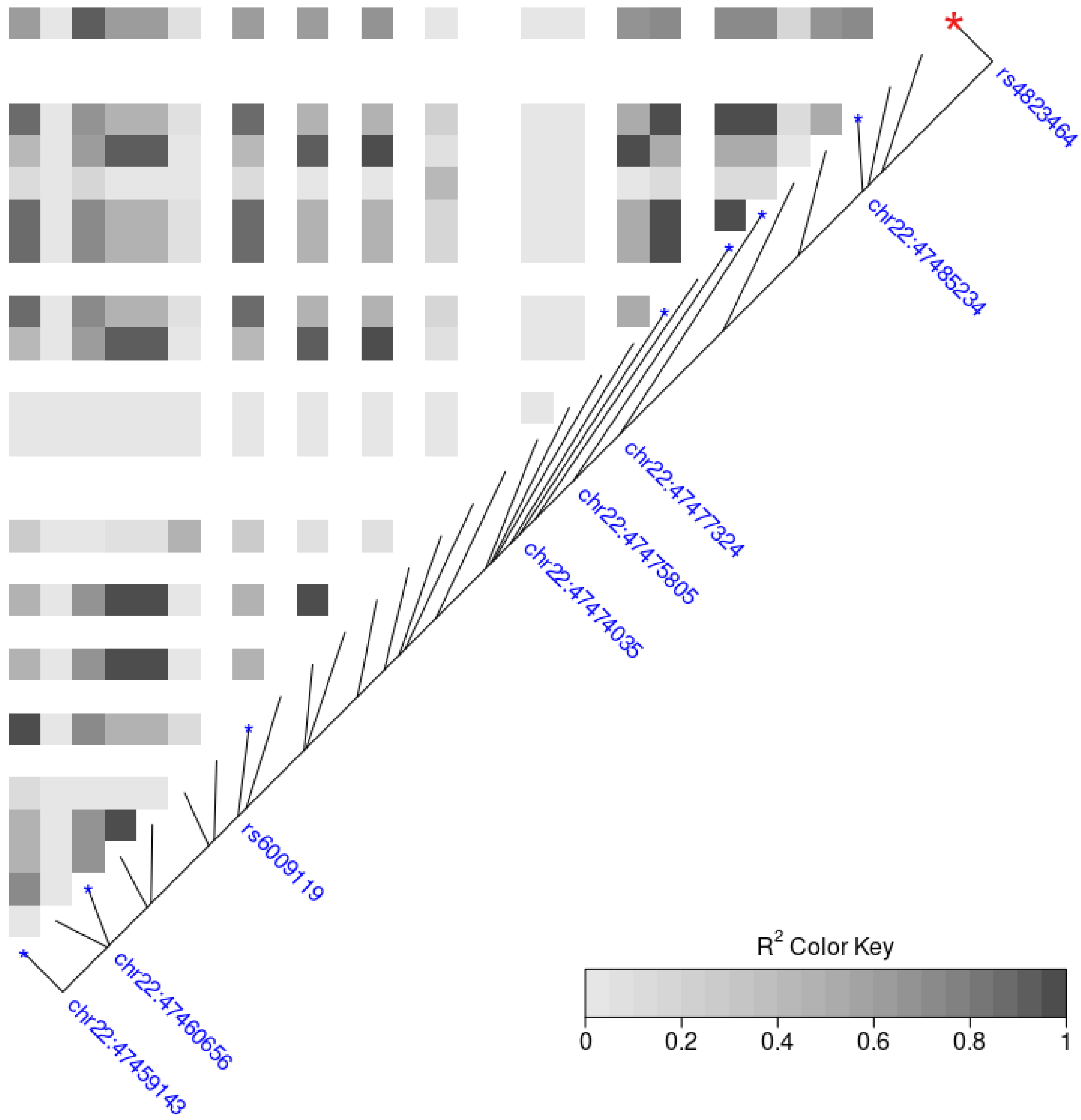

Figure S10. The LD heatmap of the genome region involving LD-block of 8 SNPs including the disease one (rs4823464). The SNPs of LD-block are marked by blue color, the disease SNP is marked by red asterisk.

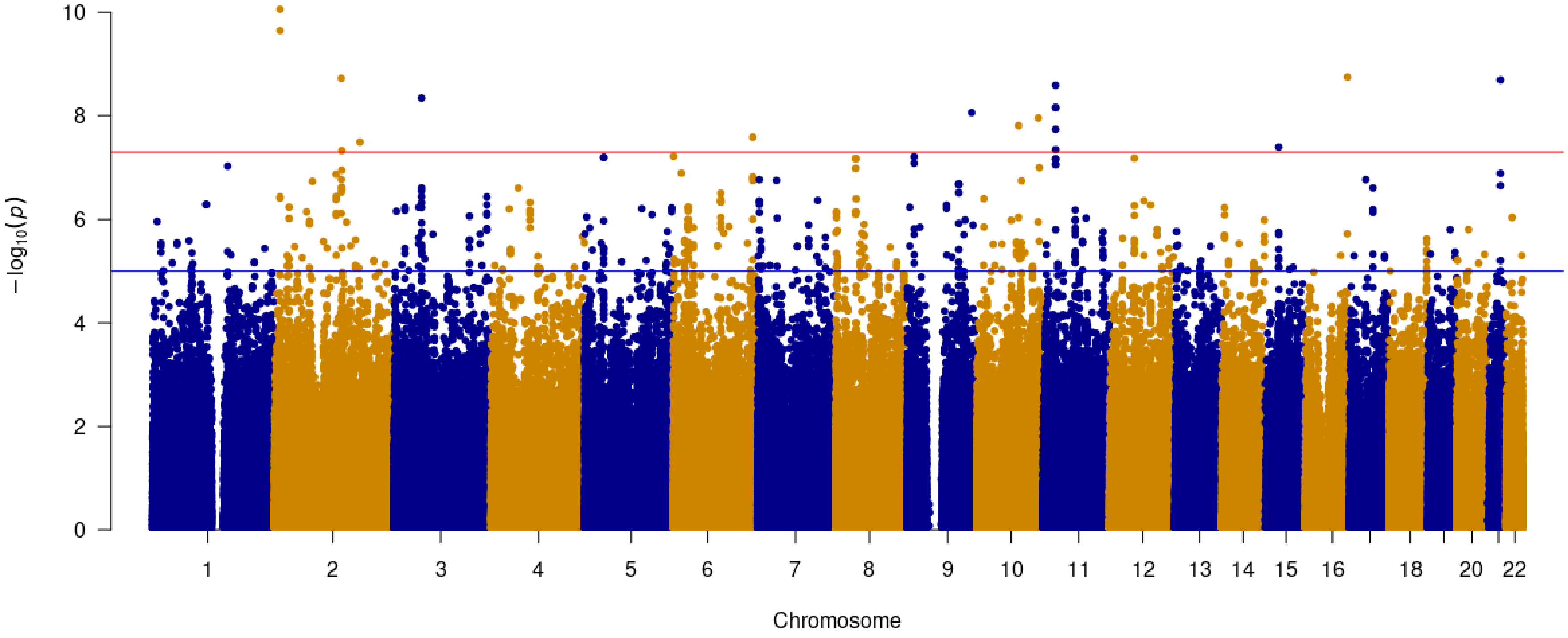

Figure S11. The Manhattan Plot with the results of classical GWAS made for allelic model

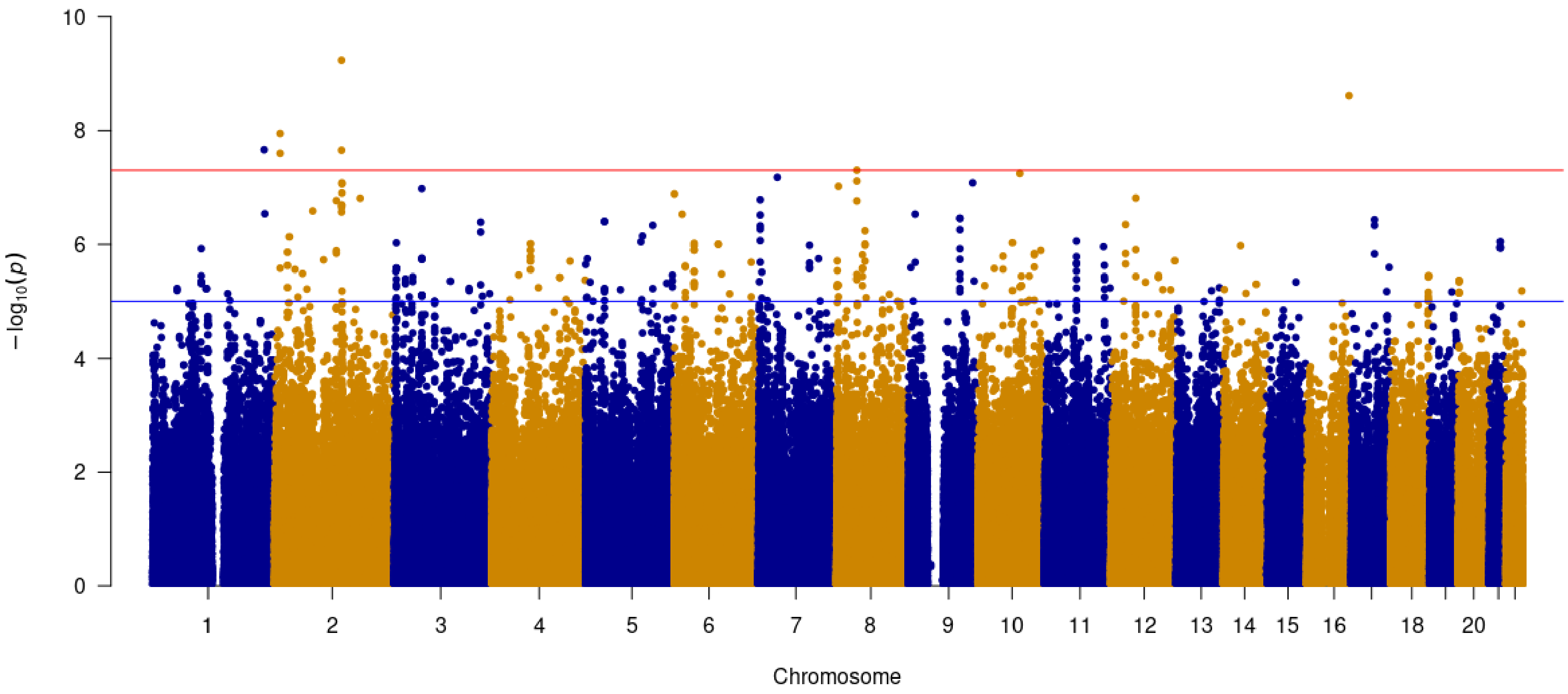

Figure S12. The Manhattan Plot with the results of classical GWAS made for dominant model

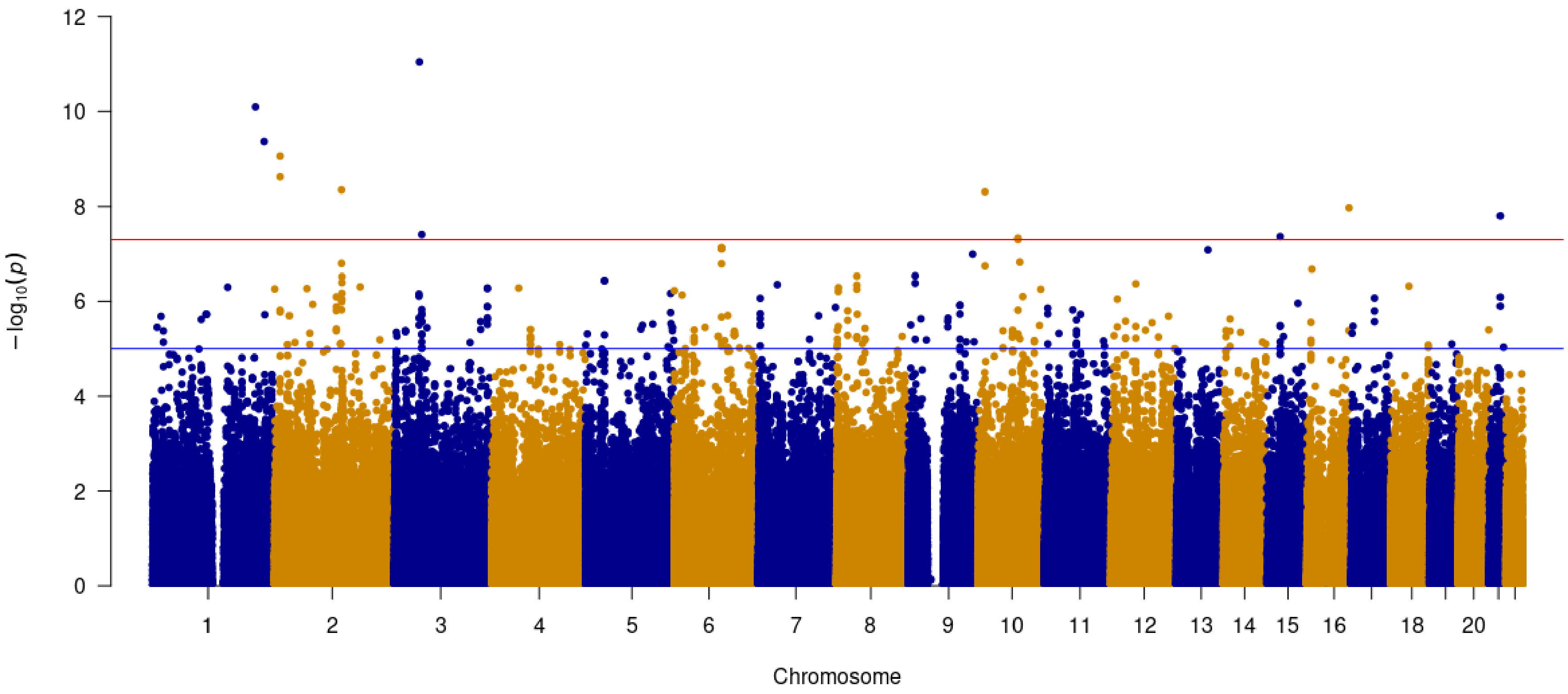

Figure S13. The Manhattan Plot with the results of classical GWAS made for genotypic model

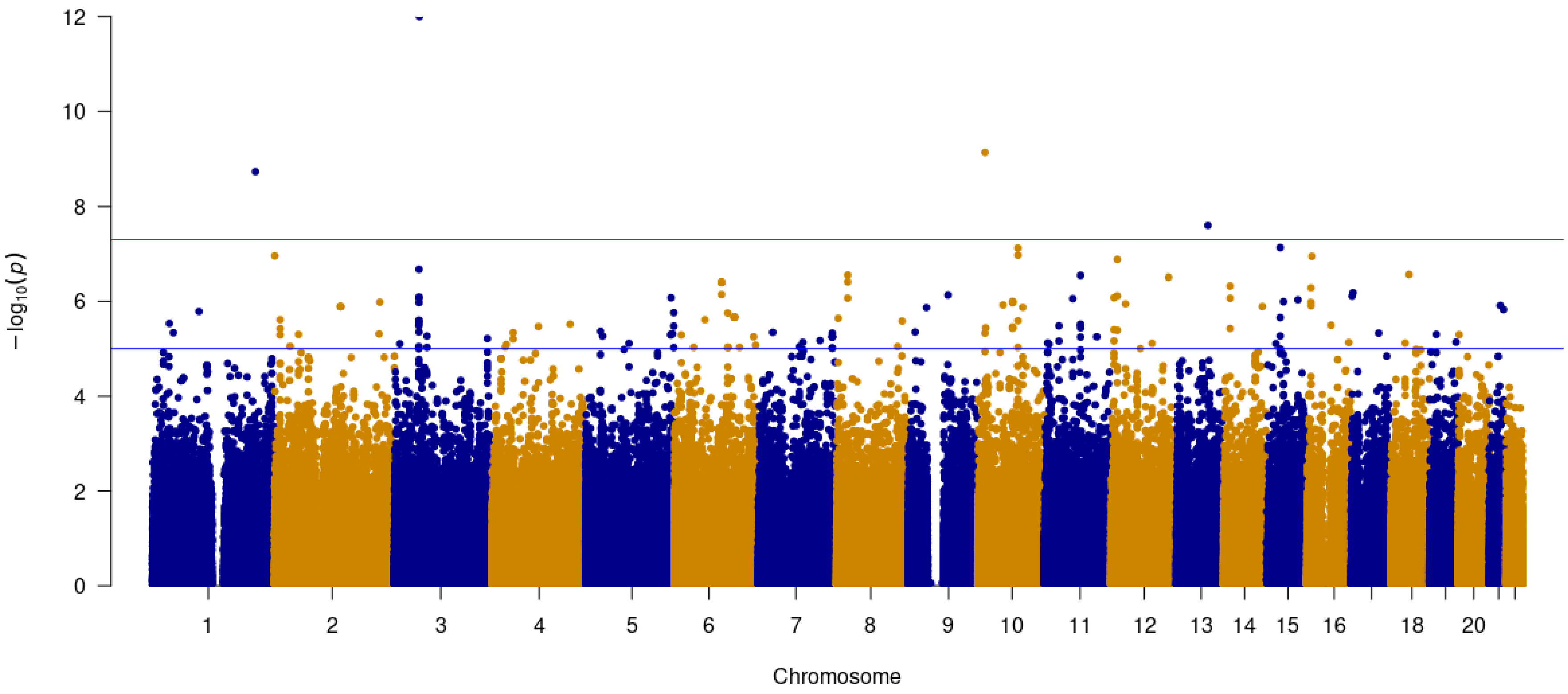

Figure S14. The Manhattan Plot with the results of classical GWAS made for recessive model

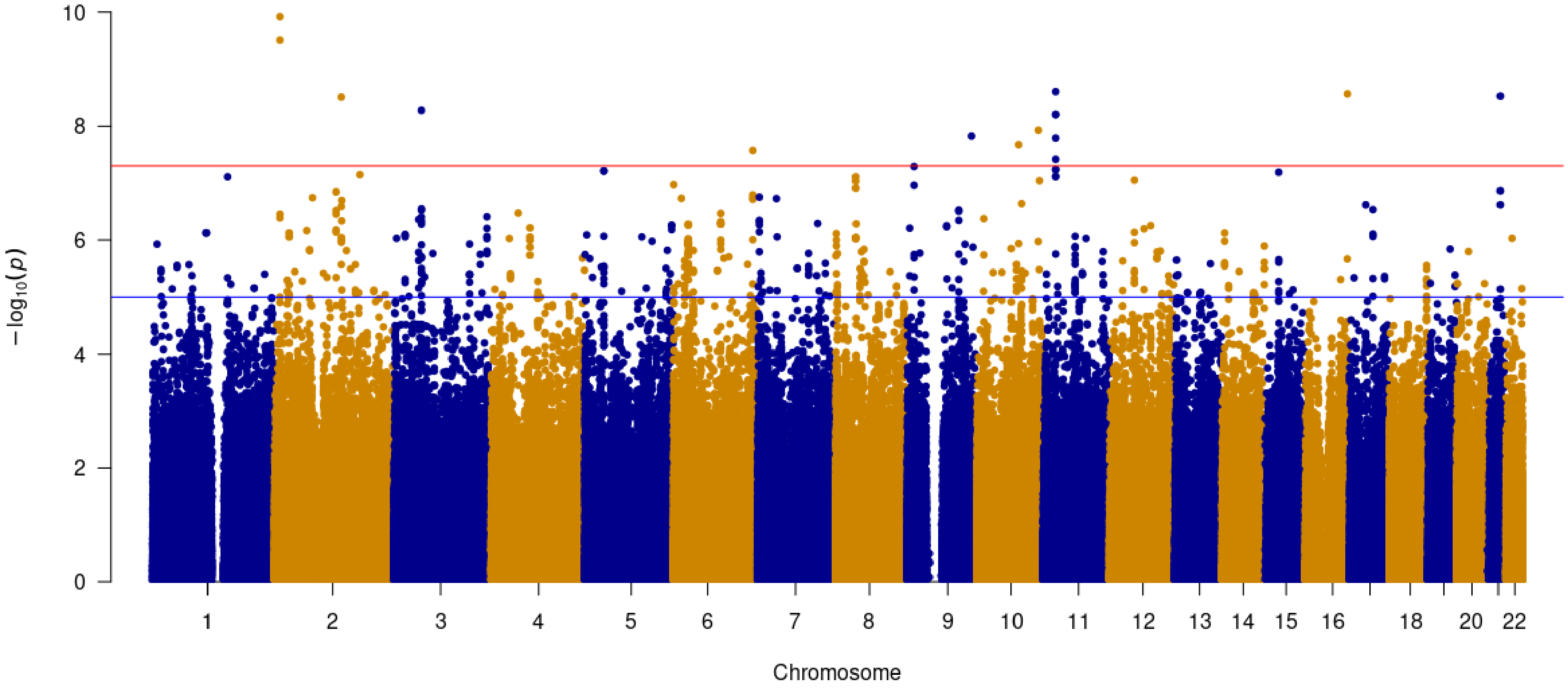

Figure S15. The Manhattan Plot with the results of classical GWAS made with Cochran-Armitage trend test

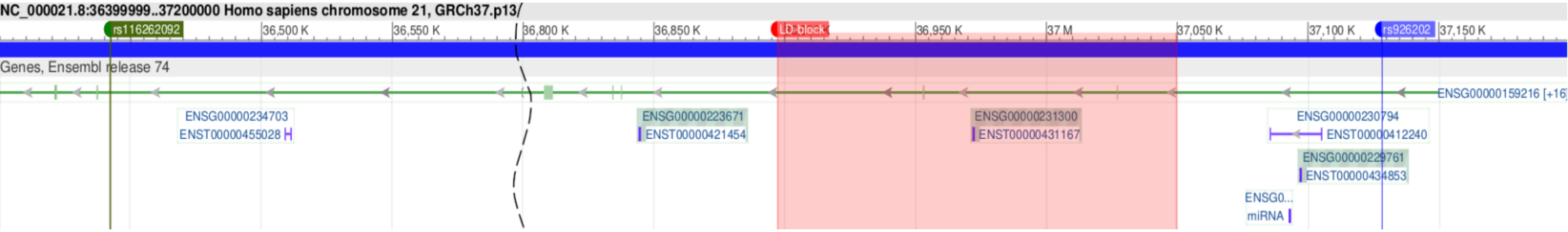

Figure S16. The gene *RUNX1* (ENSG00000159216) contains intron variants rs116262092 (21:36442465) and rs926202 (chr21:37128336) as well as the LD-block (21:36897326-37049608).

Note: For scaling a part of genomic region was cut off. Black dashed line depicts the junction of two parts of the chromosome.

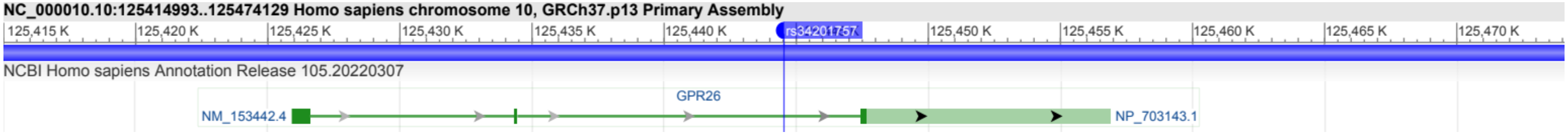

Figure S17. The gene *GPR26* contains the intron variant rs34201757

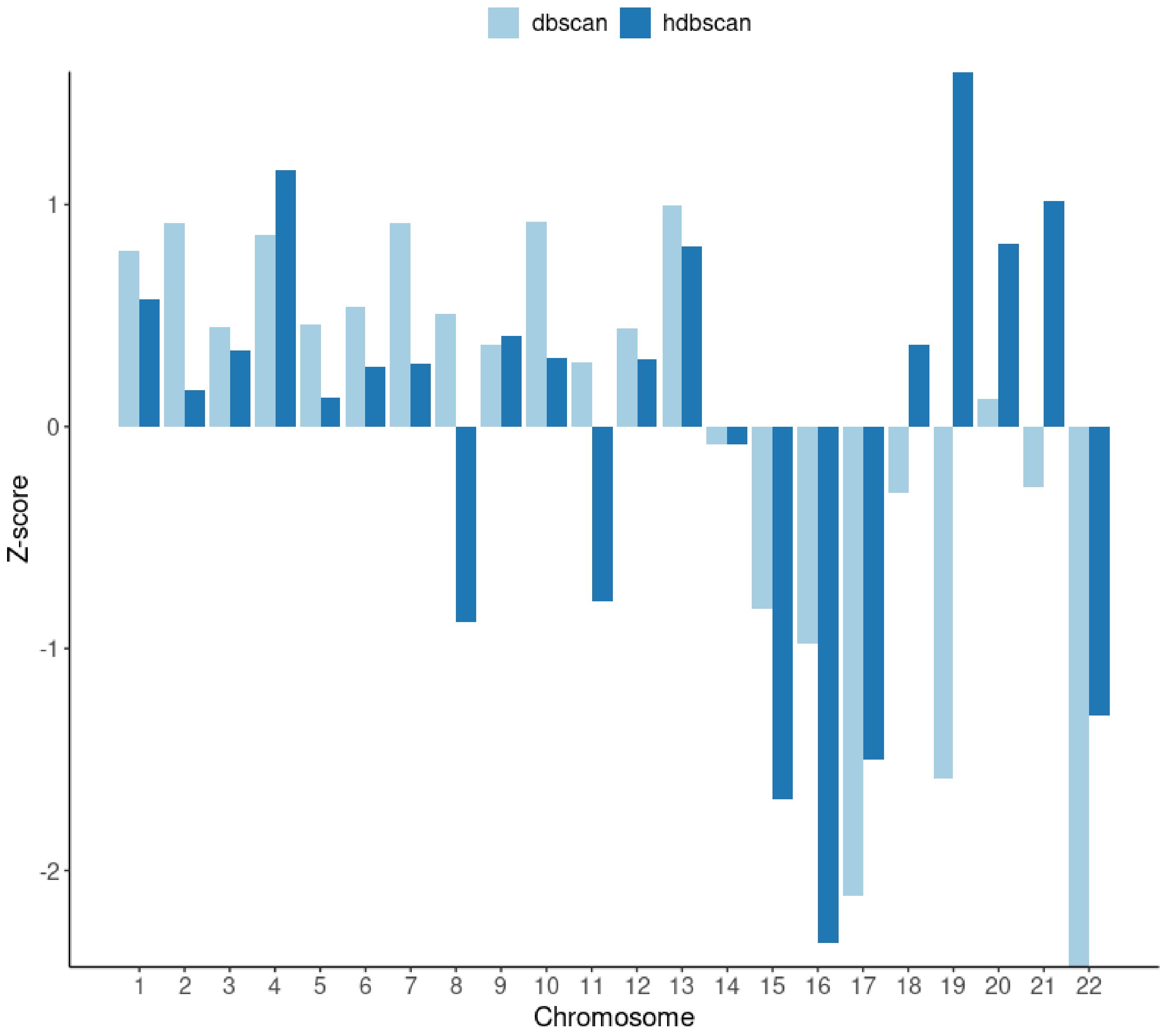

Figure S18. The z-score for the number of clusters

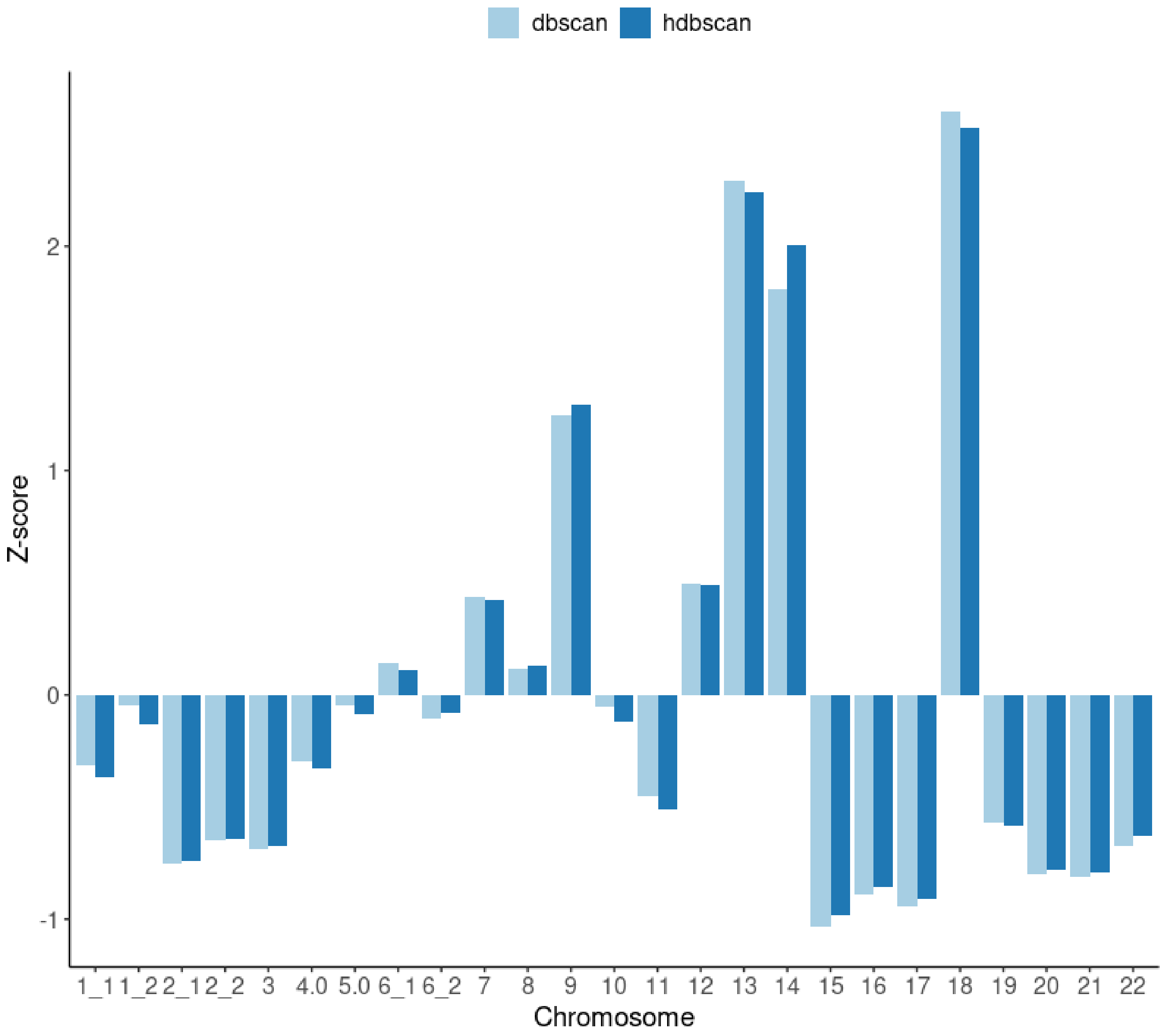

Figure S19. The z-score for Silhouette coefficient

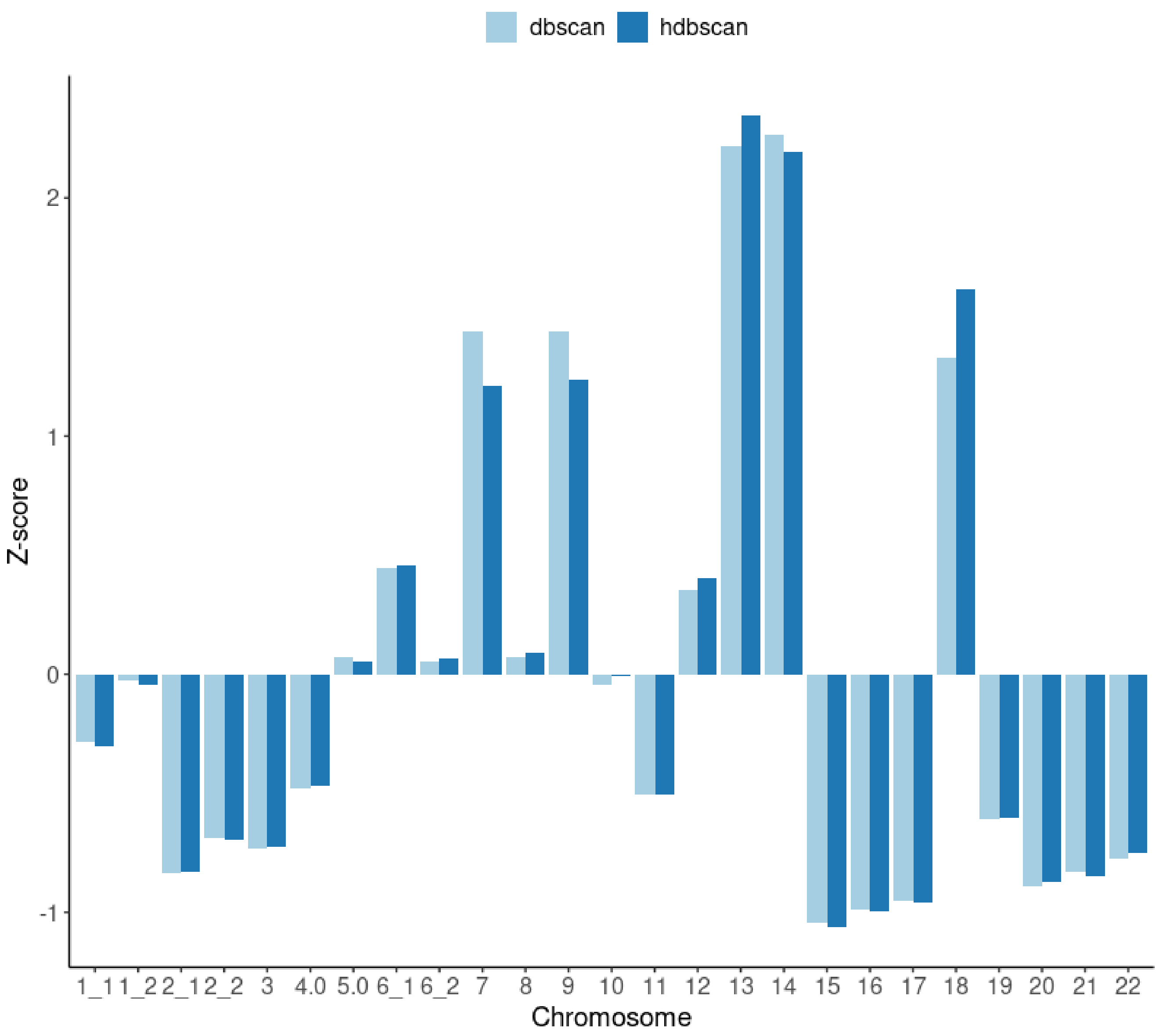

Figure S20. The z-score for Calinski and Harabasz score

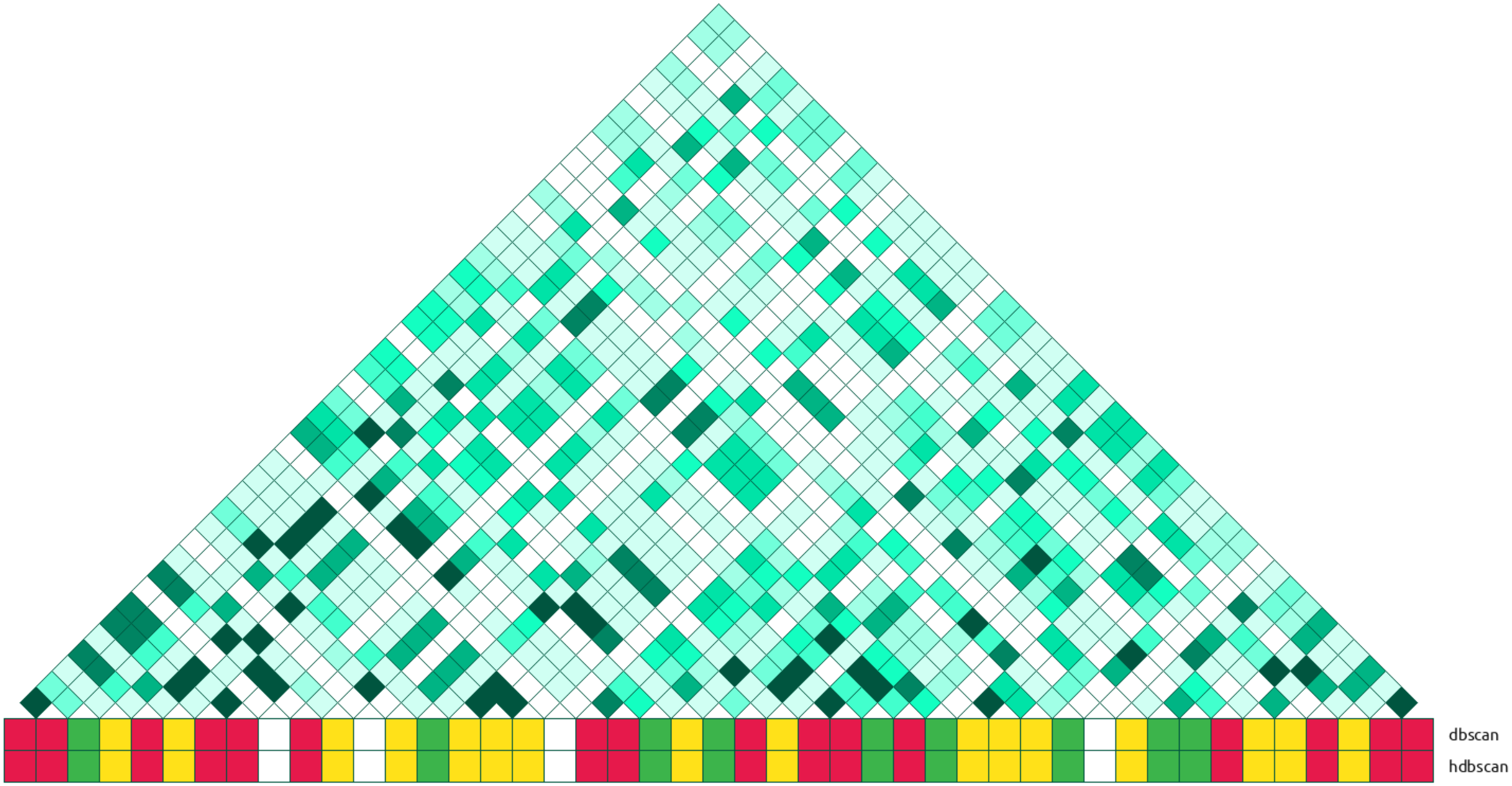

Figure S21. The LD-heatmap and cluster memberships obtained by two clustering algorithms. The region chr21:36897326-37049608. Note: The LD-block significantly associated to IS composed of 16 SNP marked by red color.

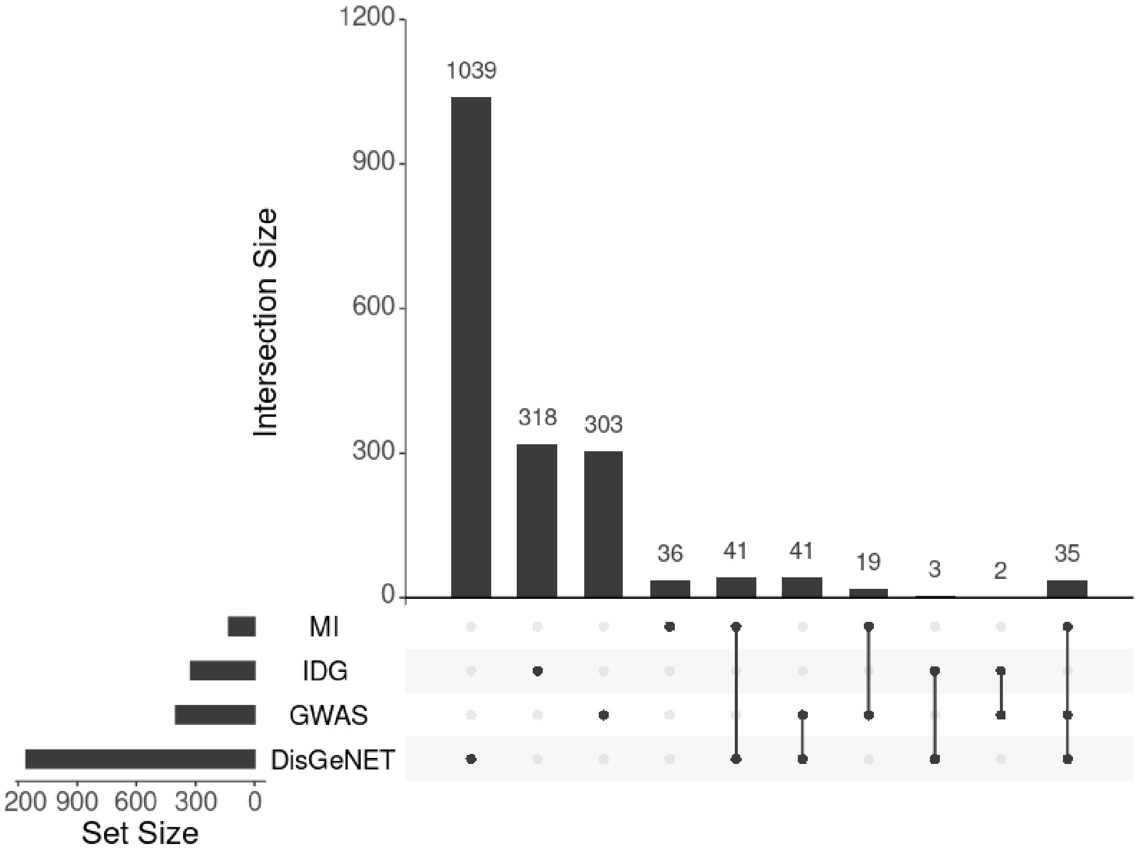

Figure S22. The sizes of gene sets known to be associated with IS and the sizes of intersections o these gene lists.

Note: MI – Monarch Initiative, IDG – Illuminating the Druggable Genome, DisGeNET – disgenet.org, GWAS – GWAS Central project.

How to read the UpSet plot, please, refer to the original article presenting the UpSetR package [1].

Supplementary files

Table_S1.xls contains two lists of LD-blocks associated to IS that were obtained with DBSCAN and HDBSCAN

Table_S2.xls contains the significant results of classical GWAS applied to simulated dataset. The tab ‘chi-square’ shows the results of statistical tests and the tab ‘logistic_regression’ shows the results of logistic regression.

Table_S3.xls has SNPs significantly associated to IS obtained with the classical GWAS.

Table_S4.xls contains the results of annotation of 29 significant SNPs with snpEff 5.1 program.

Table_S5.xlsx contains 35 genes and loci that can be considered as the risk ones.

Table_S6.xls contains three lists of genes associated to IS. Two of them were identified with DBSCAN and HDBSCAN. The third one has genes that are common to both methods.

References

1. Conway, J.R.; Lex, A.; Gehlenborg, N. UpSetR: An R Package for the Visualization of Intersecting Sets and Their Properties. Bioinformatics 2017, 33, 2938–2940, doi:10.1093/bioinformatics/btx364.
